# Effect of Personalized Prebiotic and Probiotic Supplements on the Symptoms of Irritable Bowel Syndrome: An Open-Label, Single-Arm, Multicenter Clinical Trial

**DOI:** 10.3390/nu16193333

**Published:** 2024-10-01

**Authors:** Nozomi Matsuura, Masaya Kanayama, Yuta Watanabe, Hirokazu Yamada, Loukia Lili, Akira Torii

**Affiliations:** 1 Institute of Health Science, Health Science Business Division, Kirin Holdings Company, Limited, Fujisawa, Kanagawa 251-8555, Japan; nozomi_matsuura@kirin.co.jp (N.M.); yuta_watanabe@kirin.co.jp (Y.W.); 2 Soiken Inc., Chiyoda, Tokyo 101-0052, Japan; 3 EviPRO Co., Ltd., Chiyoda, Tokyo 101-0032, Japan; 4 Thorne HealthTech, Inc., New York, NY 10019, USA; 5 Torii Medical Clinic, Setagaya, Tokyo 157-0066, Japan

**Keywords:** irritable bowel syndrome, personalized, microbiome, prebiotics, probiotics

## Abstract

Background/Objectives: Prebiotics and probiotics have been reported to improve symptoms of irritable bowel syndrome (IBS). Nevertheless, the effects of prebiotics/probiotics can vary depending on the IBS subtypes. The purpose of this study was to investigate the effects of personalized prebiotic and probiotic supplements based on intestinal microbiota and IBS subtypes in patients. Methods: Patients with diarrhea-type IBS (IBS-D), constipation-type IBS (IBS-C), and mixed-type IBS (IBS-M) were enrolled (n = 40 per group; total: n = 120). Personalized prebiotic and probiotic supplements were determined according to the IBS subtypes and intestinal microbiota. The patients received supplements for 4 weeks. The primary outcome was the change in the IBS-severity scoring system from baseline to week 4. Results:The IBS-severity scoring system significantly decreased in all patients (−38.0 [95% confidence interval (CI): −53.6, −22.4]; *p* < 0.001), in patients with IBS-D (−44.5 [95% CI: −70.6, −18.5]; *p* = 0.004) and IBS-C (−51.2 [95% CI: −79.4, −22.9]; *p* = 0.002), but not in those with IBS-M (−20.0 [95% CI: −48.0, 8.1]; *p* = 0.47). In this study, no serious adverse events were observed that had a causal relationship with the intervention. Conclusions: In conclusion, personalized prebiotic and probiotic supplements selected according to individual intestinal microbiota and IBS subtype may alleviate the severity of IBS symptoms, particularly in patients with IBS-C and IBS-D.

## 1. Introduction

Irritable bowel syndrome (IBS) is a disorder characterized by recurrent abdominal pain. This disorder is associated with a change in bowel habits; however, it is not explained by detectable structural and biochemical abnormalities in the gut [1,2]. IBS is classified into the following subtypes according to the predominant bowel habits: diarrhea-type IBS (IBS-D), constipation-type IBS (IBS-C), mixed-type IBS (IBS-M), and unclassified IBS [2]. A meta-analysis that included 423,362 subjects reported a global IBS prevalence of 3.8% (95% confidence interval [CI]: 3.1–4.5%) based on the Rome IV criteria [3]. IBS impacts daily activities, work, and quality-of-life (QOL) [4,5]; therefore, relieving symptoms is crucial.

At present, the pathophysiology of IBS is not fully elucidated. Nonetheless, it is well established that multiple factors are involved in this process, with the intestinal microbiome considered one of the most important factors [6,7]. Previous research has shown an association between intestinal microbiota and IBS. For example, a systematic review of 24 observational studies identified specific bacteria associated with IBS, such as *Bifidobacterium* and *Faecalibacterium* [8]. An observational study that involved 942 patients with IBS-D, IBS-C, and unclassified IBS reported 101 subtype-specific gut microbiome signatures [9]. Furthermore, other observational studies showed specific intestinal microbiota profiles linked to the severity of IBS [10,11].

Recent research findings have demonstrated that intestinal microbiota could emerge as a promising therapeutic target. Probiotics and prebiotics have gained significant attention as effective modulators of the intestinal microbiota, with numerous reports highlighting their potential benefits. For example, several randomized controlled trials (RCTs) involving patients with IBS who received probiotic formulations have indicated an improvement in the severity of IBS symptoms [12,13,14,15,16,17]. RCTs for prebiotics or symbiotics also showed a reduction in the severity of IBS symptoms [18,19]. In these studies, while most patients experienced relief from IBS symptoms, only a proportion responded adequately to the prebiotic/probiotic interventions [20]. A recent meta-analysis also suggested that some combinations of probiotics may be beneficial in IBS. However, the evidence was characterized by low certainty regarding the efficacy of some probiotics, moderate certainty regarding the benefit of *Escherichia* for global symptoms, and low certainty regarding the benefit of *Saccharomyces cerevisiae* for abdominal pain [21]. This variability in response may be attributed to individual differences in the profile of the intestinal microbiota [22].

Recently, personalized interventions and treatments tailored to the characteristics of individual patients have been attracting attention. Regarding dietary interventions for IBS, previous studies have indicated that a personalized diet based on intestinal microbiota improved IBS symptoms [23]. This evidence demonstrated that tailoring prebiotic and probiotic interventions to the intestinal microbiota profile of each patient is crucial for an effective IBS treatment. Interestingly, different prebiotic and probiotic formulations may have varying effects depending on the subtype of IBS [24].

However, to our knowledge, research on the impact of personalized prebiotic and probiotic treatments on IBS symptoms is limited. The purpose of this study was to investigate the effects of personalized prebiotic and probiotic supplements tailored to specific IBS subtypes and intestinal microbiota profiles on IBS symptoms.

## 2. Materials and Methods

### 2.1. Study Design

The “Trial for Application of IndividuaLized ORal prebiotics and probiotics supplements on the subjective severity in patients with Irritable Bowel Syndrome” (TAILOR-IBS) was a multicenter, open-label, non-randomized, single-arm clinical study conducted at 21 medical institutions (16 clinics and five hospitals) (Supplementary Table S1) in Japan from September 2021 to November 2023. All study procedures were conducted in accordance with the ethical standards stipulated in the Declaration of Helsinki and approved by the Japan Physicians Association Clinical Research Review Board (approval number: JPA003-2107-01). Due to the abolition of the Japan Physicians Association Clinical Research Review Board, this study was also approved by the Certified Clinical Research Review Board of Toho University (approval number: THU22005). TAILOR-IBS was registered at the Japan Registry of Clinical Trials (jRCT; registration number: jRCTs031210343). All eligible patients provided written informed consent prior to the intervention. To avoid bias and ensure quality, data collection, data management, monitoring, and statistical analyses were performed by a third-party entity (Soiken Inc., Osaka, Japan).

**Figure 1:**
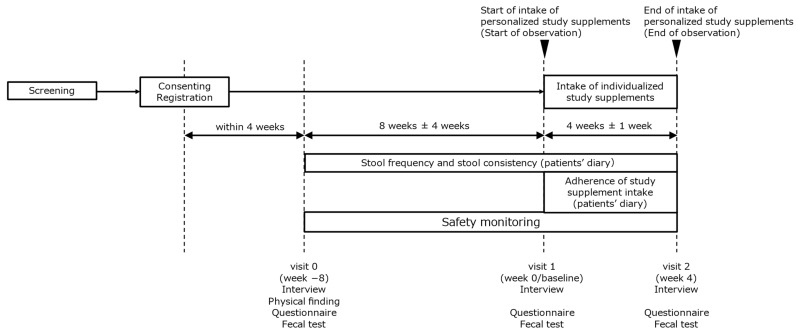
Study design.

In this study, all patients received the supplementation for 4 weeks from week 0 (baseline) to week 4 (endpoint). Three visits were scheduled for weeks −8, 0, and 4. During each visit, all patients provided stool samples to determine their intestinal microbiota profile and completed specific questionnaires for IBS symptom assessment. Information regarding demographic characteristics, IBS status, and treatment were collected at the first visit (week −8). In addition, during the study period, all patients recorded their bowel habits on a daily basis (i.e., stool frequency and stool consistency). Figure 1 shows the study design, and the observation schedule and items are shown in Supplementary Table S2.

### 2.2. Patient Population

Patients with IBS were recruited from outpatients of 21 medical institutions. The detailed inclusion/exclusion criteria are shown in Supplementary Table S3. Briefly, enrolled participants included patients diagnosed with IBS-D, IBS-C, or IBS-M based on the Rome IV diagnostic criteria [2,25] and aged 20–60 years. Patients diagnosed with gastrointestinal disorders (e.g., inflammatory bowel disease) or those with a history of major gastrointestinal surgery (e.g., gastrectomy, gastrointestinal suture, or intestinal resection) were excluded. Patients who received antibiotics or antimicrobial agents within the past 3 months prior to providing consent were also excluded.

### 2.3. Intervention

Prebiotic and probiotic supplements were personalized according to the microbiome profile of each patient. The prebiotic supplements were classified into five types, namely C1, C2, D1, D2, and M2. They were provided to patients in powder form (7500 mg of the dietary fibers arabinogalactans, partially hydrolyzed guar gum, pectin, degreasing rice bran, inulin, corn starch, and psyllium). The probiotic supplements were classified into two types, namely type B and type L, which represent a *Bifidobacterium* or *Lactobacillus* blend. They were provided in capsules of 155 mg per capsule of either *Bifidobacterium lactis* HN019 and *Bifidobacterium lactis* Bi-07 (type B) or *Lactobacillus acidophilus* NCFM and *Bacillus* coagulants SANK70258 (type L). Further details on the composition of each supplement can be found in Supplementary Table S4. These prebiotic and probiotic supplements were manufactured and supplied by Sankyo Co., Ltd. (Shizuoka, Japan).

As personalized supplements, only one type of the five prebiotic supplements (C1, C2, D1, D2, and M2) was assigned to each patient, depending on the individual profile. One or two types of probiotic supplements (types B and L) were provided; in some cases, supplements were not provided. We offered type B, type L, and both types to those who had a relatively low abundance of genus *Bifidobacterium*, genus *Lactobacillus*, and both genera, respectively. The prebiotic and probiotic supplementation decision algorithm was provided by Thorne HealthTech, Inc. (New York, NY, USA). To determine the precise probiotic and prebiotic combination, the algorithm derived information from both the reported IBS subtype and the microbiome composition profile at the DNA level collected at week −8, as extensively described in a previous study [26].

Following the algorithmic determination of the personalized combinations of prebiotic and probiotic formulations, the final supplements were approved and provided by a physician at week 0. All patients were required to consume their personalized supplements for the 4-week study period (±1 week). During this period, patients were instructed to adhere to the supplementation protocol and lifestyle, avoid adding, discontinuing, or changing the dosage or type of supplementation, and avoid major modifications of their dietary habits.

### 2.4. Study Outcomes

The primary outcome was a change in the severity of IBS symptoms, as measured by the IBS severity scoring system (IBS-SSS) from week 0 to week 4. IBS-SSS is a validated self-reported questionnaire [27,28] assessing the severity of gastrointestinal symptoms. The questionnaire assesses the following four IBS-related symptoms through five items namely abdominal pain (two items), abdominal distension (one item), bowel habit (one item), and IBS-related QOL (one item). Each item is designed to provide a score ranging from 0 to 100. The total combined score of all items of the questionnaire ranges from 0 to 500. Scores <175, 175–300, and >300 denote mild, moderate, and severe symptoms, respectively [27].

Secondary outcomes were changes in each of the IBS-SSS five-item scores from week 0 to week 4 and changes in bowel habits (i.e., stool frequency and stool consistency) and the fecal microbiome. Bowel habits were evaluated based on the daily records. The stool frequency was calculated as the 7-day average of the frequency of daily bowel movements. The stool consistency was assessed with the Bristol stool scale [29,30] and calculated as the 7-day average score. For assessment of the intestinal microbiome composition, the relative abundance of the fecal microbiome was employed as a metric.

In this study, the following intestinal microbiome was selected as a secondary outcome according to previous findings [8,31]: family *Lachnospiraceae*, genus *Bifidobacterium*, *Alistipes*, *Veillonella*, *Bacteroides*, *Eubacterium*, and *Lactobacillus*, species *Akkermansia muciniphila* and *Faecalibacterium prausnitzii*. The detailed study outcomes are shown in Supplementary Table S5.

The patients were closely monitored throughout their participation (from week −8 to week 4) for the occurrence of adverse or untoward medical events (e.g., worsening of pre-existing underlying diseases or complications).

### 2.5. Fecal Sample Collection

Fecal samples from each patient were collected with a fecal sampling kit, including guanidine thiocyanate solution (TechnoSuruga Laboratory Co., Ltd., Shizuoka, Japan), transported by mail, and stored at 4 °C until use.

### 2.6. DNA Extraction

DNA extraction was performed at LSI Medience Co., Ltd. (Tokyo, Japan). DNA was extracted from 0.2 mL of human fecal samples in guanidine thiocyanate solution using the ISOSPIN Fecal DNA kit (NIPPON GENE Co., Ltd., Tokyo, Japan). Based on the protocol, bead beating was performed thrice at 6.0 m/s for 100 s, with final solubilization of the DNA in 50 μL of eluate. DNA concentration was determined using a NanoDrop Spectrophotometer ND-1000 (Thermo Fisher Scientific Inc., Wilmington, DE, USA). The samples were stored at −80 °C until use.

### 2.7. Library Preparation and Sequencing

Library preparation and sequencing were conducted by Thorne HealthTech, Inc. DNA libraries were prepared using the Nextera XT DNA Library Preparation Kit (Illumina, Inc., San Diego, CA, USA). Genomic DNA was fragmented using a proportional amount of Illumina Nextera XT fragmentation enzyme. Unique dual indices were added to each sample, followed by 12 cycles of polymerase chain reaction to construct the libraries. DNA libraries were purified using AMpure magnetic Beads (Beckman Coulter, Inc., Indianapolis, IN, USA) and eluted in QIAGEN EB buffer (QIAGEN N.V., Venlo, The Netherlands). Subsequently, they were quantified using a Qubit 4 fluorometer and Qubit™ dsDNA HS Assay Kit (Thermo Fisher Scientific Inc.). Thereafter, they were sequenced on an Illumina NextSeq platform to produce 5–6 M reads with 2 × 150 bp per sample.

### 2.8. Data Analysis

Data pre-processing and quality control were performed as previously described [32]. Sequencing quality was assessed by FastQC [33], and human aligning reads were filtered by alignment to a human reference by Burrows–Wheeler Alignment-maximal exact matches [34]. Taxonomic classification of filtered reads was performed with KrakenUniq [35] using a database of all complete sequences of bacteria, archaea, viruses, fungi, as well as commonly used plasmid and vector sequences from the National Center for Biotechnology Information, and decontaminated eukaryotic pathogens from the Eukaryotic Pathogen Genomics Database [36].

### 2.9. Sample Size Calculation

The sample size calculation was based on the notion that any change ≥50 points in the IBS-SSS scores is considered a clinically meaningful improvement [27]. Since the change in the IBS-SSS scores in the symbiotics group was 104.3 ± 88.2 in a previous study using symbiotics [19], the standard deviation (SD) of changes in the IBS-SSS scores in this study was assumed to be 88.2. This study planned to monitor any change in IBS-SSS scores for each IBS subtype. Thus, for a two-sided test with a statistical power of 80%, it was calculated that the minimum sample size required to achieve a significance level of 0.01667 would be 36 patients per IBS subtype (Bonferroni’s adjustment). Assuming a withdrawal rate of approximately 10%, the planned sample size was set at 120 patients (i.e., 40 patients per IBS subtype).

### 2.10. Statistical Analysis

The primary and secondary outcomes were analyzed using data from the full analysis set, which included all enrolled patients but excluded those with serious protocol violations (no provision of consent, enrollment beyond the enrollment period, etc.). For the primary outcome, a sensitivity analysis was conducted using the per-protocol set, which excluded patients who did not meet the eligibility criteria, received prohibited agents and treatments, or exhibited poor adherence to the supplement intake (i.e., <75% or >120% of planned intake). Safety assessment was conducted using the safety analysis set, which included all patients who were enrolled in this study and received part of or all the study supplements. All outcomes, except for safety assessment, were analyzed in all patients and the subgroups of IBS subtypes.

Initially, the change in IBS-SSS from week 0 to week 4 was analyzed using the one-sample *t*-test. Considering the multiplicity, this analysis was first performed in all patients. Subsequently, if statistical significance was confirmed, the subgroup analysis by IBS subtype was conducted. In the subgroup analysis, Bonferroni adjustment was applied to correct for multiplicity.

The changes in each item score (five items) of the IBS-SSS from week 0 to week 4 were analyzed using the one-sample *t*-test. Changes in bowel habits (stool frequency and stool consistency) and the abundance of the fecal microbiome between week 0 and week 4 were analyzed using the Wilcoxon signed-rank test. In analyses of secondary and exploratory outcomes, adjustment for multiple testing was not applied.

Data in this study were presented as the mean (±SD or 95% CI) or median (the first quartile, the third quartile) for continuous variables and percentages for categorical variables. Unsubmitted data were treated as missing data. All *p*-values were two-sided, and a *p*-value <0.05 denoted statistical significance. All statistical analyses were performed by Soiken Inc. using SAS version 9.4 (SAS Institute Inc., Cary, NC, USA).

## 3. Results

### 3.1. Baseline Patient Characteristics

**Figure 2:**
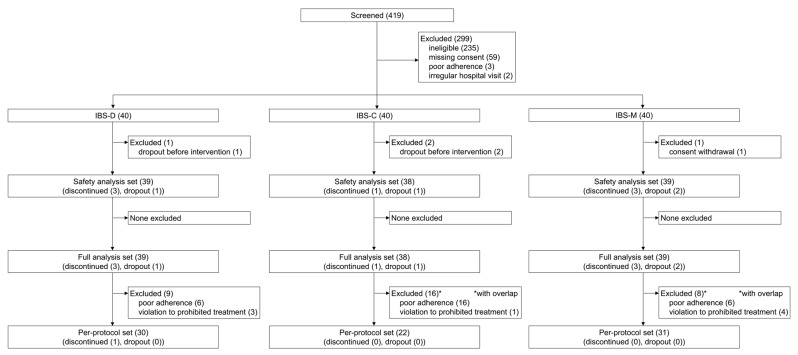
The study flow chart shows patient enrollment, allocation, and analysis. From the full analysis set to the per-protocol set, patients with poor adherence (i.e., the proportion of intake of the personalized supplement was <75% of planned) or those who violated the protocol regarding treatment (i.e., added or discontinued their medication or supplements during the observation period) were excluded. IBS, irritable bowel syndrome; IBS-C, constipation-type IBS; IBS-D, diarrhea-type IBS; IBS-M, mixed-type IBS. For the * in figure = with overlap.

Overall, 419 patients were evaluated for eligibility; finally, 120 patients with IBS-D, IBS-C, and IBS-M (n = 40 per subtype) were enrolled (Figure 2). Among patients with IBS-D, one patient dropped out due to the discontinuation of hospital visits, and three patients discontinued intake of personalized supplements (one patient due to an adverse event and two patients due to dislike of the taste of the supplement). Among patients with IBS-C, one patient dropped out due to the discontinuation of hospital visits, and one patient discontinued intake of personalized supplements due to the occurrence of an adverse event. Among patients with IBS-M, two patients dropped out due to consent withdrawal, and three patients (one patient due to an adverse event and two patients due to poor adherence to supplement intake [one overlap with dropout case due to consent withdrawal thereafter]) discontinued intake of personalized supplements.

The baseline characteristics of all patients are shown in Table 1. The mean (±SD) age was 40.3 ± 11.5 years, and most patients (76.7%) were female. The mean (±SD) duration of IBS was 6.5 ± 9.0 years, and 72.4% of patients were using pharmacological agents for IBS, whereas 38.8% were using other supplements. Interestingly, the proportions of patients who used prebiotics (1.7%) or probiotics (9.5%) were relatively low.

Table 2 shows the results of the primary outcome analysis. In all patients, the score of IBS-SSS was significantly decreased from week 0 to week 4 (−38.0 [95% CI: −53.6, −22.4]; *p* < 0.001). In the subgroup analysis, the score of IBS-SSS was also significantly decreased in patients with IBS-D (−44.5 [95% CI: −70.6, −18.5]; *p* = 0.004) and IBS-C (−51.2 [95% CI: −79.4, −22.9]; *p* = 0.002), but not in those with IBS-M (−20.0 [95% CI: −48.0, 8.1]; *p* = 0.47). Sensitivity analysis using the per-protocol set also yielded similar results; the score of IBS-SSS was significantly decreased from week 0 to week 4 in all patients, and patients with IBS-D and IBS-C; nevertheless, it was not significantly decreased in those with IBS-M (Supplementary Table S6).

### 3.2. Secondary Outcome

Table 3 shows the change in each item’s score for IBS-SSS. In all patients, unlike the score of abdominal pain frequency (*p* = 0.13), the scores of abdominal pain intensity (*p* = 0.007), abdominal bloating (*p* = 0.006), bowel habit dissatisfaction (*p* = 0.031), and daily life interference (*p* < 0.001) were significantly decreased. In the subgroup analysis, the scores of abdominal pain intensity and abdominal bloating were significantly decreased in patients with IBS-D and IBS-C but not in those with IBS-M. Scores of abdominal pain frequency and bowel habit dissatisfaction did not change significantly in any of the IBS subtypes. Nevertheless, the scores of daily life interference were significantly decreased in all IBS subtypes.

Table 4 shows the results regarding stool frequency and stool consistency. In all patients, no significant changes were observed. Moreover, stool frequency and stool consistency did not change significantly in patients with IBS-D and IBS-M; however, stool frequency increased significantly in those with IBS-C (*p* = 0.018).

**Figure 3:**
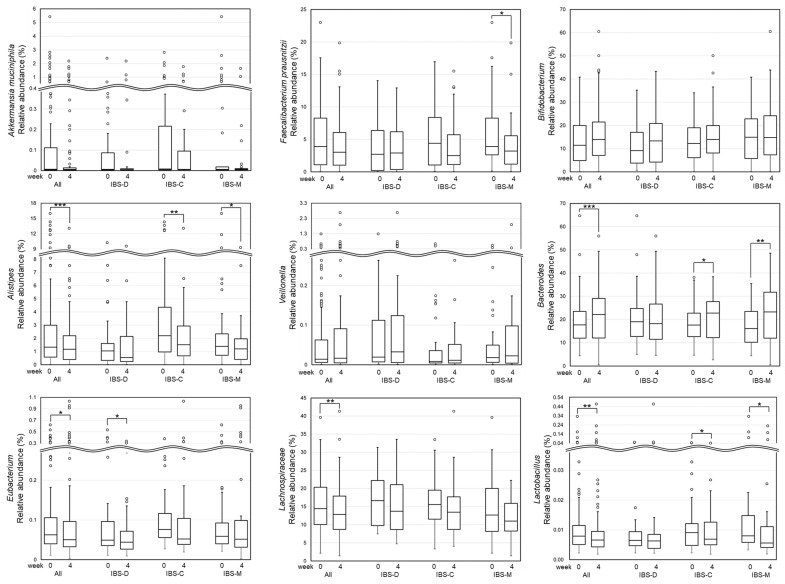
Relative abundance of fecal bacteria. The Wilcoxon signed-rank test was performed for intragroup comparisons from baseline to week 4. *, **, and *** represent *p* < 0.05, *p* < 0.01, and *p* < 0.001, respectively, for the intragroup comparison. Any data that lie >1.5-fold the interquartile range (IQR) below the first quartile or >1.5-fold the IQR above the third quartile are considered outliers. IBS, irritable bowel syndrome.

Figure 3 shows the results of the abundance of the fecal microbiome. In all patients, the relative abundance of *Alistipes* (median of change: −0.26%, *p* < 0.001), *Eubacterium* (median of change: −0.008%, *p* = 0.041), *Lachnospiraceae* (median of change: −1.39%, *p* = 0.004), and *Lactobacillus* (median of change: −0.001%, *p* = 0.003) was significantly decreased, whereas that of *Bacteroides* was significantly increased (median of change: 2.28%, *p* < 0.001). In patients with IBS-D, the abundance of *Eubacterium* was significantly decreased (median of change: −0.011%, *p* = 0.021). In patients with IBS-C, the abundance of *Alistipes* (median of change: −0.51%, *p* = 0.004) and *Lactobacillus* (median of change: −0.001%, *p* = 0.030) was significantly decreased, whereas that of *Bacteroides* (median of change: 4.91%, *p* = 0.032) was significantly increased. In patients with IBS-M, the abundance of *Faecalibacterium prausnitzii* (median of change: −0.83%, *p* = 0.027), *Alistipes* (median of change: −0.27%, *p* = 0.010), and *Lactobacillus* (median of change: −0.002%, *p* = 0.022) was significantly decreased, whereas that of *Bacteroides* (median of change: 5.34%, *p* = 0.001) was significantly increased.

### 3.3. Safety

The adverse events that occurred during the study period are summarized in Table 5. There was no occurrence of death in this study. Adverse events occurred in 27.6% of patients; however, a serious adverse event occurred only in one patient (hospitalization due to coronavirus disease-2019 [COVID-19]; 0.9%). The attending physician judged that there was no causal relationship between the intervention in this study (intake of personalized prebiotic/probiotic supplements) and the serious adverse event (COVID-19). The most frequent adverse event was COVID-19, which occurred in five patients (4.3%). Regarding the gastrointestinal symptoms, abdominal bloating (n = 4, 3.4%), constipation (n = 3, 2.6%), abdominal pain (n = 2, 1.7%), diarrhea (n = 2, 1.7%), and loose stool (n = 2, 1.7%) were reported, all of which were non-serious.

## 4. Discussion

This TAILOR-IBS study demonstrated that personalized supplementation of prebiotics/probiotics improved the severity of IBS symptoms. Particularly, IBS-SSS scores were significantly improved in patients with IBS-D and IBS-C but not in those with IBS-M. Abdominal pain intensity and abdominal bloating were significantly improved in all patients, as well as those with IBS-D and IBS-C. Daily life interference was significantly improved in all IBS subtypes. Stool frequency was significantly increased in patients with IBS-C.

Previous evidence has revealed that prebiotic [18] and probiotic [12,13,14,15,16,17] or symbiotic [19,37] supplementation improves gastrointestinal symptoms in patients with IBS. Regarding the assessment of the severity of the subjective symptoms of IBS using the IBS-SSS, a previous study in which symbiotics were administered to patients with IBS-D showed a significant decrease in IBS-SSS from baseline to week 4 (mean change ± SD: −104.3 ± 88.2, within the group: *p* < 0.01) [19]. Another study in which two probiotics (*Lactobacillus* and *Bifidobacterium*) were administered to patients with IBD also showed a greater decrease in the IBS-SSS from baseline to week 8 (mean change: −133.39, within the group: *p* < 0.0001) [12]. The tendency to improve the severity of IBS symptoms was consistent with the findings of these previous studies and the present investigation. A limitation of this study is the smaller magnitude of reported changes in IBS symptoms. Changes in the IBS-SSS scores were −38, −44.5, −51.2, and −20.0 in all patients, patients with IBS-D, patients with IBS-C, and those with IBS-M, respectively. These changes were less pronounced than those reported in the two previous studies mentioned above. This difference may be attributed to the higher reported IBS-SSS total score at baseline in those studies (i.e., 318.1 ± 63.6 [19] and 282.68 ± 60.59 [12]). This suggests that the previous studies included many patients with severe IBS symptoms.

For comparison, in the present study, the IBS-SSS total scores were 214.5 ± 92.5, 203.3 ± 100.8, 222.6 ± 87.9, and 217.8 ± 89.4 in all patients, patients with IBS-D, patients with IBS-C, and those with IBS-M, respectively, indicating the presence of mild-to-moderate symptoms. Based on these data, the personalized prebiotic and probiotic supplements improved the severity of the subjective symptoms in IBS patients with relatively mild-to-moderate symptoms. Furthermore, the improvement in the IBS-SSS scores from week 0 to week 4 was consistent with the findings of the primary analysis (full analysis set) and the sensitivity analysis (per-protocol set), showing robustness in the overall results.

This study demonstrated that, except for abdominal pain frequency, the remaining four items in the IBS-SSS (i.e., abdominal pain intensity, abdominal bloating, bowel habit dissatisfaction, and daily life interference) were significantly improved in all patients. Considering the IBS subtypes, abdominal pain intensity, abdominal bloating, and daily life interference were significantly improved in patients with IBS-D and those with IBS-C. This evidence suggested that the personalized supplementation of prebiotics/probiotics attenuated subjective symptom severity and improved the QOL of patients with IBS-D and IBS-C through improvement in abdominal pain and bloating. These results are in agreement with the current literature related to abdominal pain intensity in patients with IBS (without subtype classification) [14,17] or IBS-D [13,14,16,17], abdominal bloating in patients with IBS [14] or IBS-D [16,19], bowel habit dissatisfaction in patients with IBS [14] or IBS-D [16], and daily life interference in patients with IBS [16] or IBS-D [13,16]. For abdominal pain frequency, while two previous studies in patients with IBS [14] or IBS-D [16] reported significant improvement in abdominal pain frequency, this study did not show such improvement. This might also be attributed to the severity of abdominal pain frequency at baseline. Specifically, the value of abdominal pain frequency at baseline in this study was 24.2 ± 23.6; this value is lower than that reported in the two previous studies (i.e., 29.85 ± 11.88 [14] and 48.64 ± 21.81 [12]). This relatively low frequency of abdominal pain in this study, suggesting mild-to-moderate severity, may partly explain the lack of significant improvement in the abdominal pain frequency score. In addition, several previous studies reported negative results of prebiotics on patients’ symptoms or QOL. A systematic review and meta-analysis showed that prebiotics did not improve gastrointestinal symptoms or QOL in patients with IBS or other functional bowel disorders despite the increase of bifidobacteria [38]. Another systematic review and meta-analysis also showed that seven RCT studies (involving a total of 600 patients with IBS) demonstrated no significant difference between the prebiotic and placebo groups in overall symptom improvement [39]. This variability in response to prebiotics may be attributed to individual differences in the profile of the intestinal microbiota [22]. Personalized supplementation of prebiotics/probiotics might contribute to the improvement of the severity of IBS symptoms and QOL in this study.

Although several studies reported the beneficial effects of prebiotics or probiotics in patients with IBS-C [40,41], few reports evaluated the effects of prebiotics or probiotics using IBS-SSS in patients with IBS-C. Therefore, the present results appear to be meaningful for future IBS treatment strategies using prebiotics/probiotics. Notably, only daily life interference significantly improved in patients with IBS-M. Considering the lack of improvement in other items, the improvement in QOL in patients with IBS-M may be due to the placebo effect, which was often reported in studies of IBS [42,43,44]. IBS symptoms in patients with IBS-M often vary between diarrhea and constipation. Hence, it is possible that patients did not sense the benefit of the personalized prebiotic/probiotic supplementation in this study. Further improvement of personalized prebiotic/probiotic combinations for managing both diarrhea and constipation could alleviate the symptoms of IBS-M.

Further investigation of the specific microbiome composition revealed that the gut bacteria *Faecalibacterium*, *Alistipes*, *Bacteroides*, *Eubacterium*, *Lachnospiraceae*, and *Lactobacillus* exhibited significant changes in all patients or IBS subtypes. Research has demonstrated that the intestinal microbiota of patients with IBS differs from those of healthy individuals [8]. For example, the abundance of *Lachnospira* and *Clostridium* was significantly higher in patients with IBS [45,46]. Since *Lachnospira* produces pro-inflammatory flagellin proteins [47] and degrades intestinal mucus [48], the increase in the abundance of *Lachnospira* in patients with IBS may be associated with intestinal inflammation and associated symptoms. In this context, the significant decrease in the abundance of *Lachnospira* observed in this study may contribute to the normalization of intestinal microbiota, alleviation of inflammation, and improvement of symptoms. In addition, it has been reported that the abundance of *Bacteroides* is decreased in patients with IBS [49]. *Bacteroides fragilis* exerts its anti-inflammatory effect by producing polysaccharide A, which suppresses intestinal inflammation by inducing the proliferation of interleukin-10-producing (IL-10-producing) CD4+ T cells [50]. The significant increase in the abundance of *Bacteroides* recorded in the patients of this study may aid in the suppression of intestinal inflammation and alleviate the IBS symptoms.

On the other hand, although it has been reported that the abundance of *Alistipes* is inversely correlated with IBS symptoms [51,52], a significant decrease in the abundance of *Alistipes* was noted in this study. Although the mode of action of *Alistipes* with regard to abdominal symptoms remains poorly understood, the decrease in the abundance of *Alistipes* noted in this study might affect the symptoms of IBS. Similarly, the results obtained regarding change in the abundance of *Lactobacillus* were somewhat unexpected. *Lactobacillus* was included in the probiotic supplementation (type L) utilized in this study, and an increase in the microbiome profile of patients was hypothesized. Nevertheless, the abundance of *Lactobacillus* was significantly decreased. A possible reason for this observation may be that probiotics type L were supplied to only 24 of the 120 patients included in this investigation. Therefore, changes in the relative abundance of *Lactobacillus* in these 24 patients may not be representative of changes occurring in all patients. Another possible reason is that it may have been masked by an increase in other bacteria with high relative abundance. The relative abundance of *Lactobacillus* itself in each microbiome profile was relatively small 0.01%, which subsequently led to an even smaller change in relative abundance (0.001%). On the other hand, *Bacteroides*, whose relative abundance was approximately 20%, significantly increased with the change in the relative abundance of approximately 3.5%; the change in the absolute number of *Lactobacillus* might be masked in view of the low relative abundance. Previous studies reported the usefulness of *Lactobacillus* in clinical improvement of IBS [53,54,55]. Therefore, the administration of *Lactobacillus* itself may have also contributed to the improvement in IBS symptoms.

In summary, this study evaluated the effect of personalized supplementation of prebiotics and probiotics on ameliorating IBS symptoms. The present findings showed significant improvements in the severity of IBS symptoms in all patients, patients with IBS-C and those with IBS-D. In previous studies, there was no distinction between IBS subtypes. For instance, Skrzydło-Radomańska et al. [19] recruited only patients with IBS-D, while Williams et al. [12] recruited patients with IBS. Nonetheless, in this study, we recruited patients with IBS-C, IBS-D, and IBS-M at a ratio of 1:1:1.

This study has several limitations. Firstly, because of the single-arm study design, the causal relationship between the administration of the personalized prebiotics/probiotics and the results in this study cannot be determined. The patients were aware of the personalized prebiotic/probiotic supplementation; therefore, they might have expected improvement in IBS symptoms. High response rates, even to placebo, have been reported in RCTs for IBS [42,43,44]. Hence, the possibility that the results in this study included the influence of the placebo effect cannot be denied. However, since no significant improvement was detected in IBS-M except for daily life interference, the placebo effect in this study seemed to be limited. The personalized supplementation of prebiotics/probiotics may have beneficial effects in patients with IBS-D and IBS-C. Secondly, the short intervention period of 4 weeks may be a limitation. In several previous studies involving longer intervention periods with prebiotics [18] or probiotics [13,16,17], the difference between the active prebiotics/probiotics group and the placebo group became greater with time. Longer intervention with personalized prebiotic/probiotic supplementation may augment its effect. However, this study did not assess the mid-to-long-term effectiveness of personalized prebiotic/probiotic supplementation. Hence, additional long-term trials are required in the future. Thirdly, a period of 8 weeks from the time of assessment to the time of intervention initiation was required to determine the appropriate prebiotics and probiotics. We asked patients not to alter their dietary habits or medications throughout the study period. However, considering that the gut microbiota may change during the 8-week period, a shorter period of time between the gut microbiota assessment and the initiation of the intervention is preferable. Fourthly, the majority of medical institutions that participated in this study were clinics. This may partly explain the relatively mild-to-moderate IBS symptoms of enrolled patients and the lower severity of subjective symptoms at baseline compared with previous studies. Further studies are required to investigate the effect of personalized prebiotics/probiotic supplements on the severity of subjective symptoms in patients with more severe IBS. Finally, this study only involved medical institutions in Japan and Japanese patients. Taking into account that the gut microbiota differs across countries/ethnicities [56], the generalizability of the present results is unknown. Thus, additional international studies are warranted.

The personalized prebiotics and probiotics utilized in this study were associated with a good safety profile based on an adverse event rate of 27.6% and the occurrence of only one serious adverse event (COVID-19) without a causal relationship with the intervention.

## 5. Conclusions

In this study, the use of personalized prebiotic/probiotic supplements improved the severity of IBS symptoms, especially in patients with IBS-C and IBS-D. Our findings suggest that personalized prebiotic and probiotic supplements have the potential to alleviate symptoms in patients with IBS-C and IBS-D. However, further studies are necessary to comprehensively evaluate the effect of these supplements.

## Figures and Tables

**Table 1:** Baseline characteristics.

Characteristic	All (n = 116)
Age (years)	40.3 ± 11.5
Female	89 (76.7%)
BMI (kg/m^2^)	21.3 ± 3.7
Duration of IBS (years) ^¶^	6.5 ± 9.0
Use of pharmacological agents for IBS	84 (72.4%)
Use of supplements	45 (38.8%)
Use of prebiotic supplements	2 (1.7%)
Use of probiotic supplements	11 (9.5%)

Data are presented as the mean ± standard deviation (n) for continuous variables and as the number of patients (n) and proportion (%) for categorical variables.^¶^ n = 109. BMI, body mass index; IBS, irritable bowel syndrome.

**Table 2:** Changes in the IBS-SSS from week 0 to week 4.

	Week 0	Week 4	Change from Week 0 [95% CI]	*p*-Value
All	214.5 ± 92.5 (116)	176.3 ± 98.3 (102)	−38.0 [−53.6, −22.4] (102)	<0.001
IBS-D	203.3 ± 100.8 (39)	156.1 ± 113.1 (33)	−44.5 [−70.6, −18.5] (33)	0.004
IBS-C	222.6 ± 87.9 (38)	165.2 ± 97.0 (33)	−51.2 [−79.4, −22.9] (33)	0.002
IBS-M	217.8 ± 89.4 (39)	205.1 ± 79.0 (36)	−20.0 [−48.0, 8.1] (36)	0.47

As primary analysis, changes in IBS-SSS from week 0 to week 4 were analyzed using the full analysis set. Data are presented as the mean ± standard deviation (n) for measurement and mean [95% confidence interval] (n) for change from week 0. A one-sample *t*-test was conducted for intragroup comparison. For intragroup comparisons among IBS subtypes, the *p*-value was adjusted using Bonferroni’s correction. CI, confidence interval; IBS, irritable bowel syndrome; IBS-C, constipation-type IBS; IBS-D, diarrhea-type IBS; IBS-M, mixed-type IBS; IBS-SSS, IBS-severity scoring system.

**Table 3:** Changes in each item score of the IBS-SSS from week 0 to week 4.

	Week 0	Week 4	Change from Week 0 [95% CI]	*p*-Value
All patients				
Abdominal pain intensity	37.2 ± 28.2 (116)	30.5 ± 29.1 (102)	−7.1 [−12.2, −1.9] (102)	0.007
Abdominal pain frequency	24.2 ± 23.6 (116)	21.0 ± 22.8 (102)	−2.7 [−6.2, 0.8] (102)	0.13
Abdominal bloating	36.4 ± 29.6 (116)	29.1 ± 28.5 (102)	−7.6 [−12.9, −2.2] (102)	0.006
Bowel habit dissatisfaction	58.1 ± 27.5 (116)	50.8 ± 28.1 (102)	−7.9 [−15.0, −0.7] (102)	0.031
Daily life interference	58.5 ± 29.6 (116)	44.9 ± 31.1 (102)	−12.8 [−17.9, −7.8] (102)	<0.001
Patients with IBS-D				
Abdominal pain intensity	39.1 ± 28.7 (39)	28.2 ± 29.6 (33)	−10.4 [−20.3, −0.6] (33)	0.038
Abdominal pain frequency	27.3 ± 25.3 (39)	21.8 ± 23.0 (33)	−3.5 [−10.3, 3.3] (33)	0.30
Abdominal bloating	28.2 ± 27.8 (39)	19.1 ± 25.5 (33)	−8.5 [−15.5, −1.5] (33)	0.019
Bowel habit dissatisfaction	52.8 ± 29.0 (39)	46.4 ± 34.7 (33)	−8.5 [−20.3, 3.3] (33)	0.15
Daily life interference	55.8 ± 34.2 (39)	40.6 ± 35.8 (33)	−13.6 [−21.8, −5.5] (33)	0.002
Patients with IBS-C				
Abdominal pain intensity	34.3 ± 26.3 (38)	24.0 ± 27.2 (33)	−9.8 [−15.6, −4.0] (33)	0.002
Abdominal pain frequency	23.2 ± 26.6 (38)	19.4 ± 25.3 (33)	−3.6 [−9.7, 2.4] (33)	0.23
Abdominal bloating	44.6 ± 29.3 (38)	28.3 ± 29.4 (33)	−15.8 [−26.2, −5.4] (33)	0.004
Bowel habit dissatisfaction	59.3 ± 31.4 (38)	49.3 ± 29.0 (33)	−7.8 [−24.6, 9.1] (33)	0.35
Daily life interference	61.3 ± 28.4 (38)	44.2 ± 30.1 (33)	−14.2 [−25.4, −3.0] (33)	0.015
Patients with IBS-M				
Abdominal pain intensity	38.0 ± 29.9 (39)	38.5 ± 29.3 (36)	−1.5 [−12.1, 9.1] (36)	0.78
Abdominal pain frequency	22.2 ± 18.7 (39)	21.8 ± 20.8 (36)	−1.1 [−7.3, 5.1] (36)	0.72
Abdominal bloating	36.6 ± 30.2 (39)	39.1 ± 27.6 (36)	0.8 [−9.0, 10.7] (36)	0.86
Bowel habit dissatisfaction	62.4 ± 21.1 (39)	56.4 ± 19.0 (36)	−7.4 [−16.5, 1.8] (36)	0.11
Daily life interference	58.6 ± 26.3 (39)	49.3 ± 27.5 (36)	−10.8 [−18.4, −3.3] (36)	0.006

Data are presented as the mean ± standard deviation (n) for measurements and mean [95% confidence interval] (n) for change from week 0. A one-sample *t*-test was conducted for intragroup comparison. CI, confidence interval; IBS, irritable bowel syndrome; IBS-C, constipation-type IBS; IBS-D, diarrhea-type IBS; IBS-M, mixed-type IBS; IBS-SSS, IBS-severity scoring system.

**Table 4:** Changes in stool frequency and stool consistency from week 0 to week 4.

	Week 0	Week 4	Change from Week 0	*p*-Value
All patients				
Stool frequency (number/day)	1.1 [0.7, 1.7] (113)	1.1 [0.9, 1.7] (111)	0.1 [−0.1, 0.4] (111)	0.10
Stool consistency (Bristol scale)	4.1 [3.5, 4.9] (113)	4.1 [3.4, 4.7] (111)	0.0 [−0.7, 0.5] (111)	0.30
Patients with IBS-D				
Stool frequency (time/day)	1.7 [1.1, 2.3] (38)	1.6 [1.1, 2.6] (37)	0.1 [−0.3, 0.4] (37)	0.58
Stool consistency (Bristol scale)	4.3 [4.0, 5.1] (38)	4.4 [4.0, 4.8] (37)	0.0 [−0.6, 0.3] (37)	0.50
Patients with IBS-C				
Stool frequency (time/day)	0.9 [0.6, 1.3] (37)	1.0 [0.7, 1.4] (37)	0.1 [−0.1, 0.4] (37)	0.018
Stool consistency (Bristol scale)	3.6 [2.7, 4.3] (37)	3.6 [2.9, 4.2] (37)	−0.1 [−0.7, 0.5] (37)	0.51
Patients with IBS-M				
Stool frequency (time/day)	1.0 [0.9, 1.7] (38)	1.1 [0.9, 1.6] (37)	0.1 [−0.3, 0.3] (37)	0.78
Stool consistency (Bristol scale)	4.1 [3.7, 5.0] (38)	3.9 [3.4, 4.5] (37)	0.1 [−1.1, 0.6] (37)	0.64

Data are presented as the median [the first quartile, the third quartile] (n). Wilcoxon signed-rank test was conducted for intragroup comparison. IBS, irritable bowel syndrome; IBS-C, constipation-type IBS; IBS-D, diarrhea-type IBS; IBS-M, mixed-type IBS.Primary Outcome.

**Table 5:** Adverse events.

Severity of Adverse Event	All Patients (n=116)	
Death	0 (0.0%)	
Any adverse event	32 (27.6%)	
Any serious adverse event	1 (0.9%)	
**Type of adverse event**	**Total**	**Serious**
COVID-19	5 (4.3%)	1 (0.9%)
Abdominal bloating	4 (3.4%)	0 (0.0%)
Constipation	3 (2.6%)	0 (0.0%)
Abdominal pain	2 (1.7%)	0 (0.0%)
Anemia	2 (1.7%)	0 (0.0%)
Diarrhea	2 (1.7%)	0 (0.0%)
Fever	2 (1.7%)	0 (0.0%)
Loose stool	2 (1.7%)	0 (0.0%)
Adverse reaction for injection	1 (0.9%)	0 (0.0%)
Anal fissure	1 (0.9%)	0 (0.0%)
Asthma	1 (0.9%)	0 (0.0%)
Back pain	1 (0.9%)	0 (0.0%)
Cold sensation	1 (0.9%)	0 (0.0%)
Cystitis	1 (0.9%)	0 (0.0%)
Endometritis	1 (0.9%)	0 (0.0%)
Genital bleeding	1 (0.9%)	0 (0.0%)
Hypertension	1 (0.9%)	0 (0.0%)
Hyperthyroidism	1 (0.9%)	0 (0.0%)
Hyperuricemia	1 (0.9%)	0 (0.0%)
Insomnia	1 (0.9%)	0 (0.0%)
Intermenstrual bleeding	1 (0.9%)	0 (0.0%)
Nausea	1 (0.9%)	0 (0.0%)
Pharyngitis	1 (0.9%)	0 (0.0%)
Premenstrual syndrome	1 (0.9%)	0 (0.0%)
Seasonal allergy	1 (0.9%)	0 (0.0%)

Data are presented as number of patients (%). COVID-19, coronavirus disease-2019.

## Data Availability

The original contributions presented in the study are included in the article/Supplementary Materials, further inquiries can be directed to the corresponding author/s.

## Supplementary Materials

The following supporting information can be downloaded at: https://www.mdpi.com/article/10.3390/nu16193333/s1, Table S1: Participating medical institutions. Table S2: Observation schedule and items. Table S3: Eligibility Criteria. Table S4: Ingredients and combinations of study supplements. Table S5: Study outcomes. Table S6: The change in IBS-SSS from week 0 to week4.
