# Supplementary material for: Effect of Personalized Prebiotic and Probiotic Supplements on the Symptoms of Irritable Bowel Syndrome: An Open-Label, Single-Arm, Multicenter Clinical Trial
Source: Nutrients. 2024 Oct 1;16(19):3333. doi: 10.3390/nu16193333 (PMC11478705; doi:10.3390/nu16193333)
Supplement: Supplementary file 1 [file nutrients-16-03333-s001.zip › nutrients-3212411-supplementary.pdf]

Supplementary Table S1. Participating medical institutions.

Participating medical institution	Responsible investigator	Number of enrolled patients
Kogawa clinic	Kogawa Tetsuya	17
Tsurukamekai	Ishibashi Fumiaki	16
Tama Medical Clinic	Watanabe Nobutoshi	13
Matsushima Hospital	Nishino Haruo	10
Satoh Naika	Satoh Yasuhiro	10
Chiho Clinic	Kanemaru Chiho	9
Kawaguchi Gastroenterology Clinic	Kawaguchi Yoshiaki	8
Kawasaki Rinko Hospital	Oda Ichiro	8
Nippon Medical School Musashi Kosugi Hospital	Futagami Seiji	6
Shinjuku Tsurukame Clinic	Tanaka Ryu	5
CPC Clinic	Koshino Hideyuki	3
Fujita Hospital	Hongo Hitoshi	2
Japanese Red Cross Medical Center	Yoshida Hideo	2
Kiraku naika clinic	Tsuno Shinichi	2
Murano clinic	Murano Mitsuyuki	2
Nakamura Azabujuban Clinic	Nakamura Mitsuyasu	2
Toho University Omori Medical Center	Fujimoto Ai	2
Kajigaya Clinic	Hanyu Ken	1
Kameido Ekimae Clinic	Kanematsu Tooru	1
Matsushima Hospital	Kuromizu Joji	1
Torii Medical Clinic	Torii Akira	0

Supplementary Table S2. Observation schedule and items

	Consenting/registration	visit 0 (week -8)	visit 1 (week 0)	visit 2 (week 4)	discontinuation
1. Eligibility	○				
2. Patients' backgrounds		○			
3. Treatment information for IBS		○	○	○	△
4. Intestinal microbiota		○	○	○	△
5. Questionnaire 1		○			
6. Questionnaire 2 (IBS-SSS)		○	○	○	△
7. Stool frequency, stool consistency		←	○	→	△
8. Adherence of study supplement intake			←	○ →	△
9. Medication information other than for IBS treatment		○	○	○	△
10. Adverse event		←	○	→	

○, mandatory; △, optional

IBS: irritable bowel syndrome; IBS-SSS: irritable bowel syndrome symptom severity scale;

Supplementary Table S3. Eligibility Criteria

Inclusion criteria	Patients who meet all of the following criteria are included in this study;<br>1. Patients who are diagnosed with diarrhea-predominant irritable bowel syndrome (IBS-D), constipation-predominant irritable bowel syndrome (IBS-C), or mixed-form irritable bowel syndrome (IBS-M) based on Rome IV diagnostic criteria.<br>2. Male and female aged 20 years or older, and younger than 60 years.<br>3. Subjects whose native language is Japanese.<br>4. Subjects who provide their written consent form to participate in the study.
Exclusion criteria	Patients who meet any of the following criteria are excluded from the study;<br>1. Patients who took antibiotics or antimicrobial agents within the last 3 months before giving their consent.<br>2. Patients who are diagnosed with gastrointestinal disorders such as inflammatory bowel disease.<br>3. Patients with hisotory of major gastrointestinal surgery such as gastrectomy, gastrointestinal suture, or intestinal resection.<br>4. Patients who have taken antidepressants, antipsychotics, or anxiolytics on a daily basis within the last 3 months before giving their consent (excluding those who take the drugs as needed)<br>5. Patients who take drugs that suppress immune function at giving their consent.<br>6. Patients who take drugs that affect blood glucose or blood pressure

at giving their consent.

7. Patients with history of allergy against the active ingredients* of the study foods

*active ingredients: arabinogalactan, Guar Gum degradant, pectin, delipidated rice bran, inulin, cornstarch, psyllium, bifidobacteria, lactobacillus

8. Patients who have a history of cancer in the gastrointestinal organs or who are currently receiving treatment or medications for cancer in the gastrointestinal organs.

9. Patients with serious cerebrovascular disease, cardiac disease, hepatic disease, renal disease, gastrointestinal disease, or infectious disease requiring notification

10. Patients who are pregnant, breastfeeding, possibly pregnant, or planning to be pregnant.

11. Patients who need legal representative for giving their consent.

12. Patients who are considered to be unsuitable for participating in this study by investigators.

IBS: irritable bowel syndrome

Supplementary Table S4. Ingredients and combinations of study supplements

	Prebiotics					Probiotics	
	C1	C2	D1	D2	M2	B	L
Arabinogalactan	○	○			○		
Guar gum degradation product	○		○		○		
Pectin		○		○	○		
Defatted rice bran	○		○		○		
Inulin	○	○		○			
Corn starch			○	○			
Psyllium husk powder	○	○	○	○	○		
Bifidobacterium lactis HN019						○	
Bifidobacterium lactis Bi-07						○	
Lactobacillus acidophilus NCFM							○
Bacillus coagulans SANK 70258							○

Supplementary Table S5. Study outcomes

Primary endpoint	Change in score of IBS-severity scoring system (IBS-SSS) from baseline to week 4
Secondary endpoints	1. Change in each item score of IBS-SSS (abdominal pain intensity, abdominal pain frequency, abdominal bloating, bowel habit dissatisfaction, and daily life interference) from baseline to week 4
	2. Change in stool frequency and stool consistency from baseline to week 4
	3. Change in fecal microbiome from baseline to week 4
Exploratory endpoints	1. Change in total score of IBS-severity scoring system (IBS-SSS) from baseline to week 4 stratified by IBS subtypes
	2. Change in each item score of IBS-SSS from baseline to week 4 stratified by IBS subtypes
	3. Change in stool frequency and stool consistency from baseline to week 4 stratified by IBS subtypes
	4. Change in fecal microbiome from baseline to week 4 stratified by IBS subtypes

Supplementary Table S6. The change in IBS-SSS from week 0 to week4.

	week 0	week 4	change from week 0 [95% CI]	p value
All	206.2±94.0 (83)	169.7±96.0 (78)	-38.4 [-55.3, -21.5] (78)	<0.001
IBS-D	197.4±97.7 (30)	152.9±103.1 (28)	-48.0 [-73.8, -22.2] (28)	0.002
IBS-C	209.5±99.1 (22)	151.3±107.6 (20)	-56.3 [-95.9, -16.6] (20)	0.024
IBS-M	212.2±88.9 (31)	197.7±75.5 (30)	-17.6 [-44.9, 9.7] (30)	0.59

As sensitivity analysis, the change in IBS-SSS from week 0 to week 4 was analyzed using per-protocol set.

Data are presented as the mean ± standard deviation (n).

One-sample t-test was conducted for intragroup comparison.

For intragroup comparisons of IBS subtypes, significance was adjusted by Bonferroni’s correction.

IBS-SSS: irritable bowel syndrome symptom severity scale; IBS: irritable bowel syndrome; CI: confidence interval; IBS-D: diarrhea-type IBS; IBS-C: constipation-type IBS; IBS-M: mixed-type IBS
